# Supplementary material for: Detangling electrolyte chemical dynamics in lithium sulfur batteries by operando monitoring with optical resonance combs
Source: Nat Commun. 2023 Nov 14;14:7350. doi: 10.1038/s41467-023-43110-8 (PMC10645864; doi:10.1038/s41467-023-43110-8)
Supplement: Supplementary file 1 — Supplementary Information [file 41467_2023_43110_MOESM1_ESM.pdf]

Supplementary information

Detangling electrolyte chemical dynamics in lithium sulfur batteries by operando monitoring with optical resonance combs

Fu Liu[1,2], Wenqing Lu[3], Jiaqiang Huang[4], Vanessa Pimenta[3], Steven Boles[5], Rezan Demir-Cakan[6,7*] & Jean-Marie Tarascon[1,2,8*]

[1]Collège de France, Chimie du Solide et de l'Energie—UMR 8260 CNRS, Paris, France.
[2]Réseau sur le Stockage Electrochimique de l'Energie (RS2E)—FR, CNRS 3459, Amiens, France.
[3]Institut des Matériaux Poreux de Paris (IMAP), ESPCI Paris, Ecole Normale Supérieure, CNRS, PSL University, Paris, France
[4]The Hong Kong University of Science and Technology (Guangzhou), Sustainable Energy and Environment Thrust, Nansha, Guangzhou, Guangdong 511400, P. R. China
[5]Department of Energy and Process Engineering, Faculty of Engineering, Norwegian University of Science and Technology (NTNU), Trondheim, Norway
[6]Institute of Nanotechnology, Gebze Technical University, Kocaeli, 41400, Turkey
[7]Department of Chemical Engineering, Gebze Technical University, Kocaeli, 41400, Turkey
[8]Sorbonne Université–Université Pierre-et-Marie-Curie Paris (UPMC), Paris, France

* Correspondence: demir-cakan@gtu.edu.tr (R. Demir-Cakan); jean-marie.tarascon@college-de-france.fr (J.-M. Tarascon)

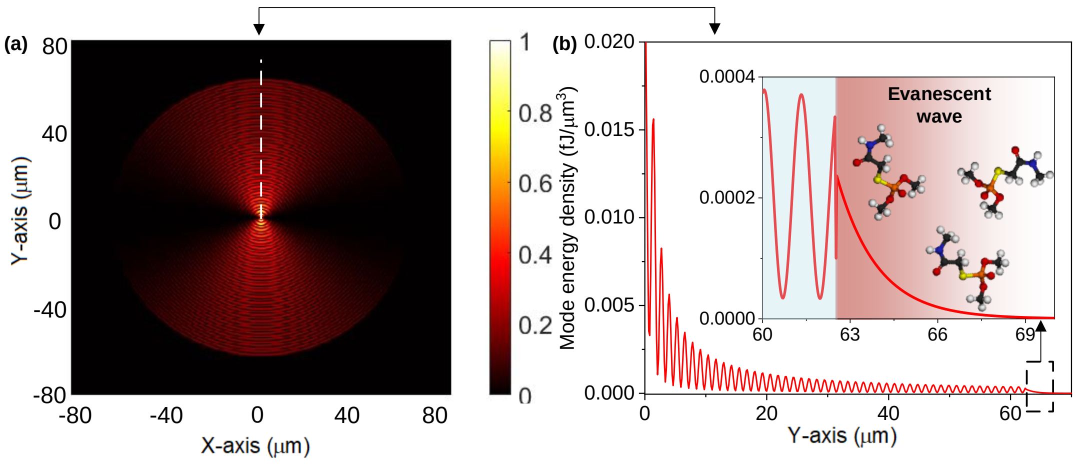

Fig. S1 | Simulation of the electric field intensity of a cladding mode. (a) Electric field intensity of mode across fiber. (b) Mode energy density distribution along fiber diameter. The inset shows the energy distribution in and around fiber surface and the chemical molecules are sensed in evanescent field region[1,2].

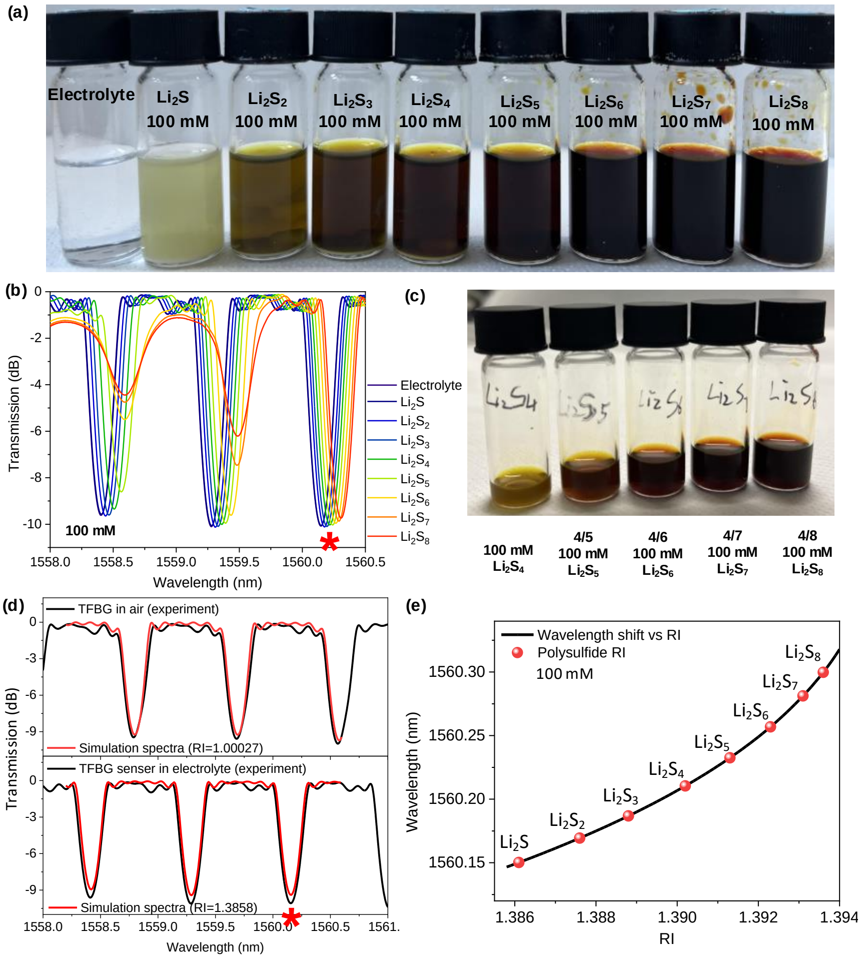

Fig. S2 | Prepared and tested polysulfide solution. (a) 100 mM Li_2S_x polysulfide solution, Li_2S_x (x=1, 2, 3, ⋯, 8). (b) TFBG cladding mode response to polysulfide of (a), the cladding modes at 1558.5 nm and 1559.25 nm shift to longer wavelength, while the amplitude

decreases for longer chain polysulfide. That is because the guided mode is transformed to leaky mode resulting from the corresponding refractive index solution being equal to or bigger than that of effective index of the guided mode, indicating the loss of their total internal reflection within the fiber. Therefore, the guided mode at 1560.2 nm wavelength is used for sensing. (c) The polysulfide solutions with the same sulfur concentration. Dilute the 100 mM Li_2S_x (x=5, 6, 7, 8) solution to ensure the sulfur concentration is 400 mM (the same as Li_2S_4). Note that even the sulfur chain length of polysulfide is different but the refractive index does not change if the sulfur concentration is the same. (d and e) RI calibration of polysulfide based on TFBG spectra simulation. First, all simulation parameters were convinced by matching the experimental and simulation spectra in air (top panel of d), which is set as "background" spectra. Second, 100 spectrum simulations are achieved by increasing surrounding RI gradually from 1.3858 to 1.3957 at 0.0001 intervals. Finally, find out the spectra matching to that of electrolyte (bottom panel of d) and polysulfide, thus the corresponding RI is obtained (e). Note that the intrinsic force to shift the wavelength of sensor is the surrounding RI variation. The basic principle is that polysulfide concentration changes the refractive index of electrolyte which shifts the wavelength of cladding mode then. The wavelength shift is not linearly relating to RI decided by intrinsic mechanism of TFBG[3], but quite linear to sulfur concentration. Thus, linear relation between wavelength shift and sulfur concentration can be built directly without bothering by refractive index.

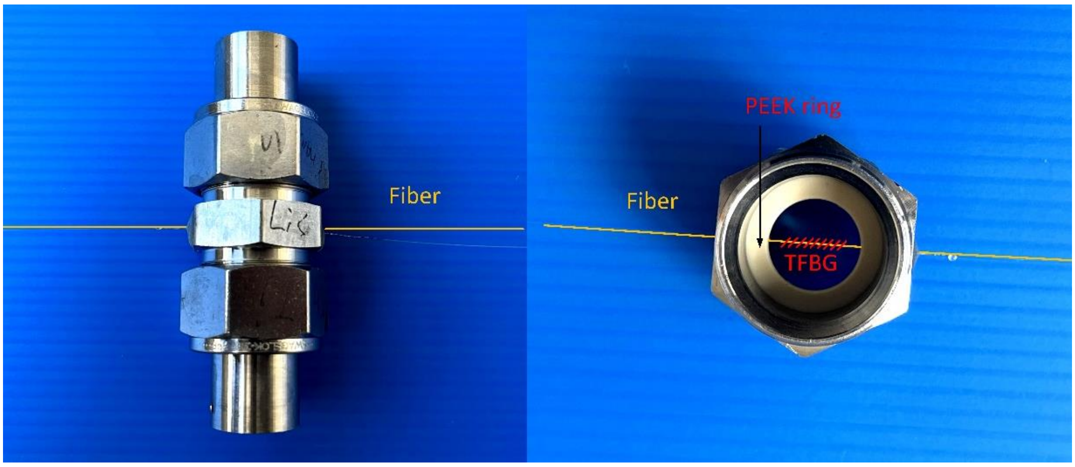

Fig. S3 | Configuration of fiber sensor integrated Swagelok.

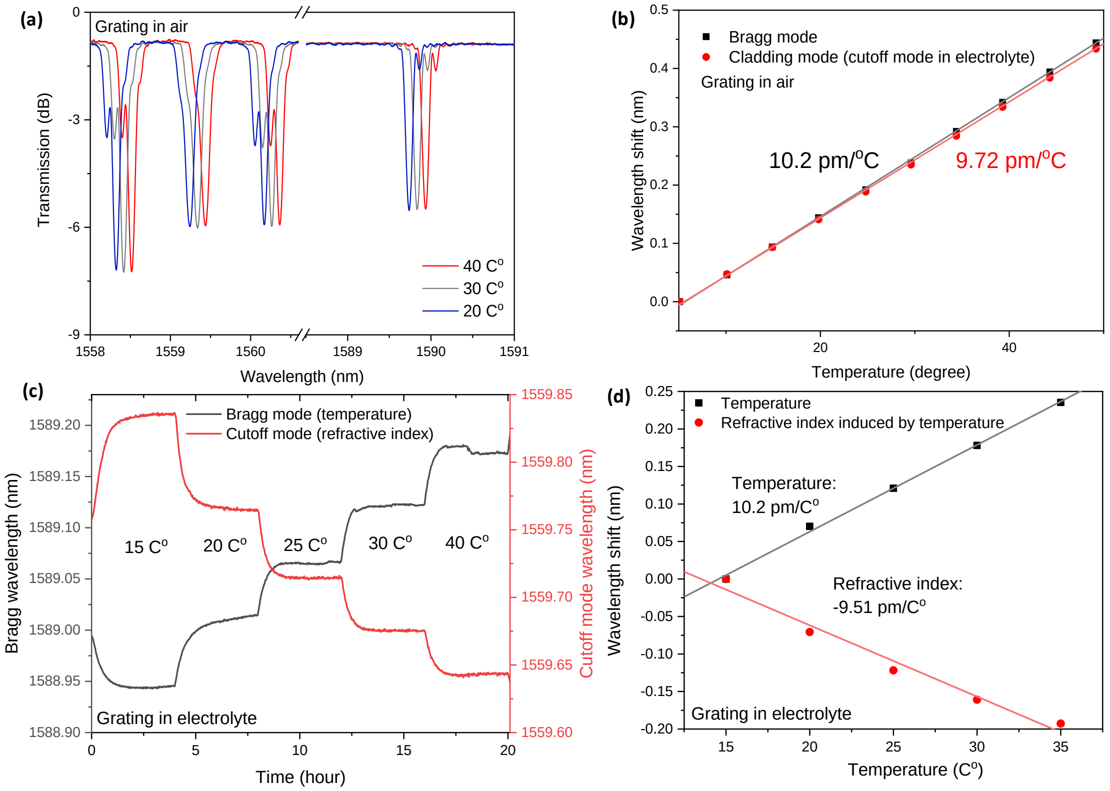

Fig. S4 | Thermal calibration of electrolyte: (a) The thermal response of spectra in air and (b) the corresponding thermal sensitivity of core mode and target cladding mode (cutoff mode in electrolyte); (c) The thermal response in electrolyte and (d) sensitivity.

The thermal effects can be totally removed by a thorough thermal calibration process that enlists several steps as follows: Step 1: by testing temperature response of fiber sensor in air (Fig. S4a,b), the thermal sensitivity of core mode is 10.2 pm/C° and target cladding mode (cutoff mode in electrolyte) is 9.72 pm/C° (cladding mode thermal sensitivity is always smaller than that of core mode)[4]. Step 2: by testing temperature response of fiber sensor immersed in electrolyte (Fig. S4c,d), the wavelength shift of the cutoff mode comprises two parts: temperature (9.72 pm/C° obtained from step 1) and the temperature-modulated refractive index of the electrolyte (-9.51 pm/C° in Fig. S4d).

When the cell is cycled with the fiber sensor, the wavelength shift will be composed of temperature, temperature-induced refractive index, and polysulfide-induced refractive index of electrolyte. By manually compensating for the thermal effects on the basis of steps 1 and 2, wavelength shift will be linked solely to the polysulfide generated.

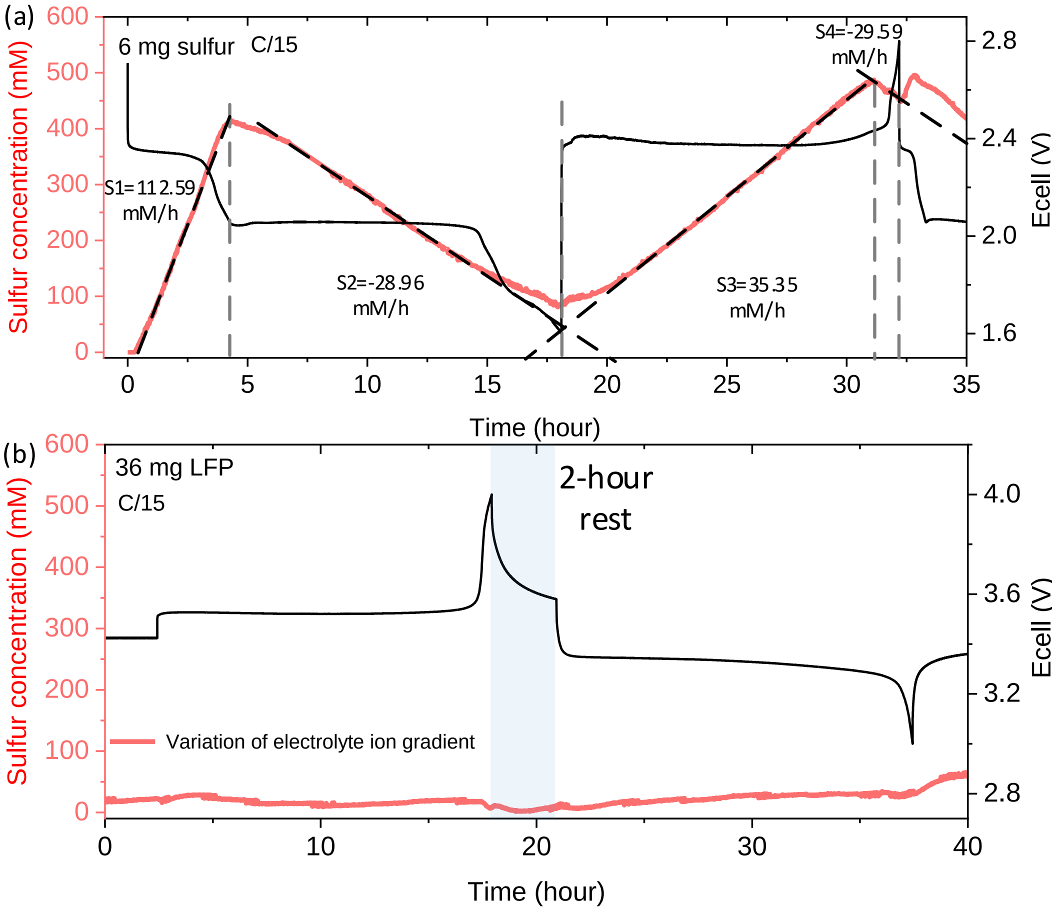

Fig. S5 | Ion transportation of electrolyte. (a) Operando measurement of LSB together with the sulfur concentration rate for each plateau. (b) Operando measurement of cell considering LFP as cathode. Note: to investigate the effect of ion concentration gradient of electrolyte including Li^+, $TFSI^-$ and NO_3^- in DOL and DME, a reference experiment considering cathode using the 36 mg LFP (much larger than 6 mg of sulfur) was carried out, which indicates that the corresponding RI of electrolyte variation is 20 times smaller than that in LSB when inducing polysulfide. Therefore, for the real case with less active material in LSB, the ion transportation of electrolyte itself can be neglected and the wavelength shift of sensor will be related to sulfur concentration. Note: $Concentration\ ratio = \left|\frac{\text{first plateau concentration slope}}{\text{second plateau concentration slope}}\right|$.

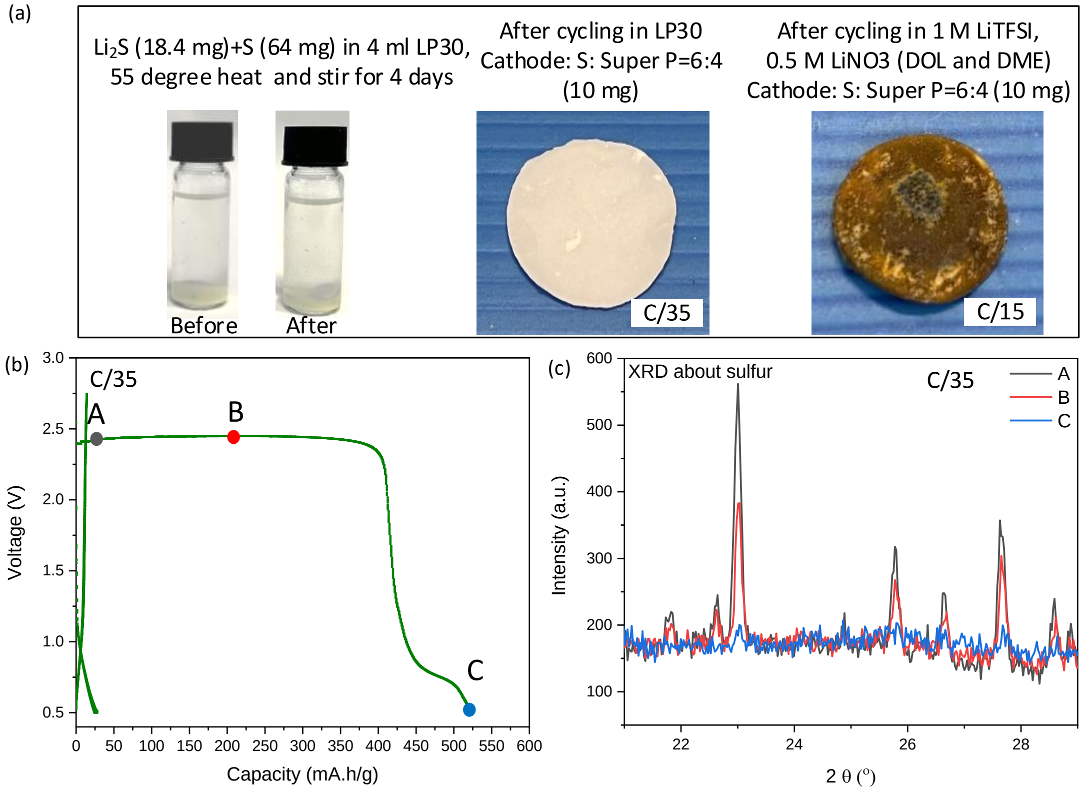

Fig. S6 | LSB using LP30 electrolyte. (a) Li_2S_6 polysulfide prepared in LP30 electrolyte by mixing Li_2S and S powder (the same method as that made in 1 M LiTFSI, 0.5 M $LiNO_3$ in DOL/DME (1:1, v/v)) demonstrating that Li_2S_6 are not formed (further confirmed by the color of separator after LSB cycling). (b) The temporal voltage (green dot) vs capacity of operando XRD test, and the corresponding XRD pattern marked by A, B, C are depicted in (c), which indicates that the solid sulfur was totally consumed by the end of first plateau to form thiocarbonate-like solid electrolyte interphase.

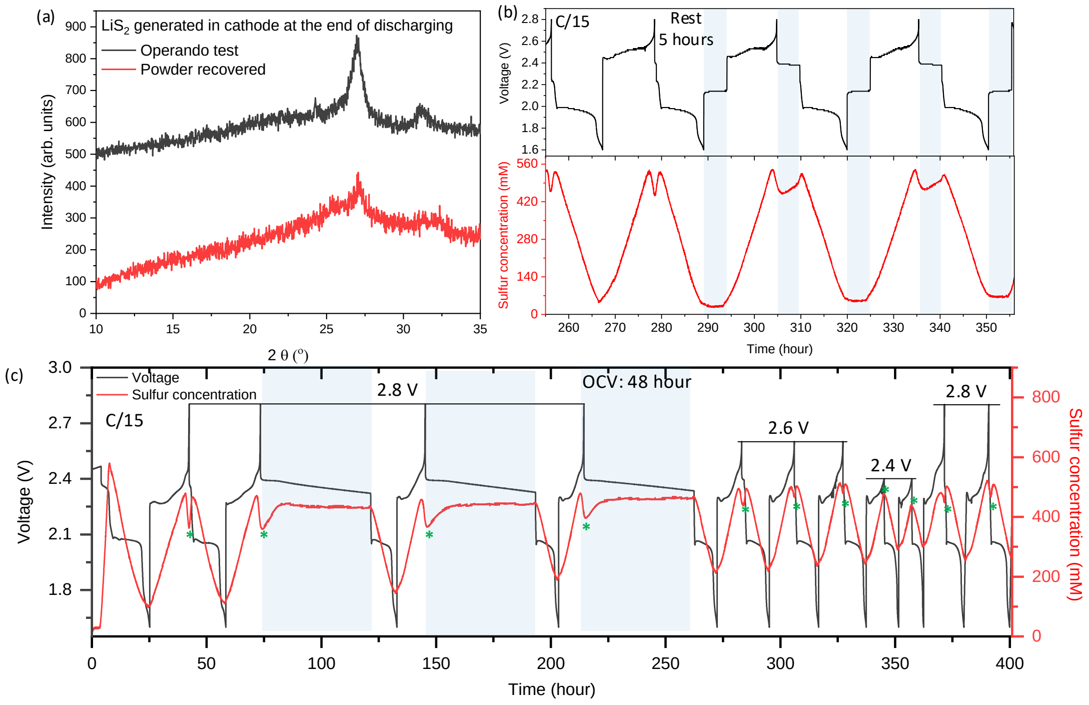

Fig. S7 | The crystallization of sulfur (Li_2S) after the charging (discharging). (a) The XRD pattern of Li_2S generated in sulfur and super P composite cathode at the end of discharge during operando test (gray line), convinced by recovering the cathode power (red line) pertaining to the fact that the $LiNO_3$ additives, used as shuttle suppressor, leads to form a protective passivation layer on lithium that prevent crystallization of Li_2S on anode[5,6]. (b) The temporal voltage (black line) and decoded sulfur concentration of electrolyte (red line) considering 5 hours rest at the end of charge and discharge, respectively, where sulfur concentration keeps a constant at the end of discharge because there is no additional chemical reaction transforming of Li_2S to soluble polysulfide, while at the end of charge the sulfur concentration increase gradually due to the comproportionation reactions that transforms recrystallized sulfur to soluble polysulfide. (c) The recrystallized sulfur governed by comproportionation reactions and potential voltage. The shaded region in blue stands for 48 hours OCV starting at the end of charge so that the re-crystallized sulfur (marked by green asterisk "*") will be dissolved to soluble polysulfide gradually.

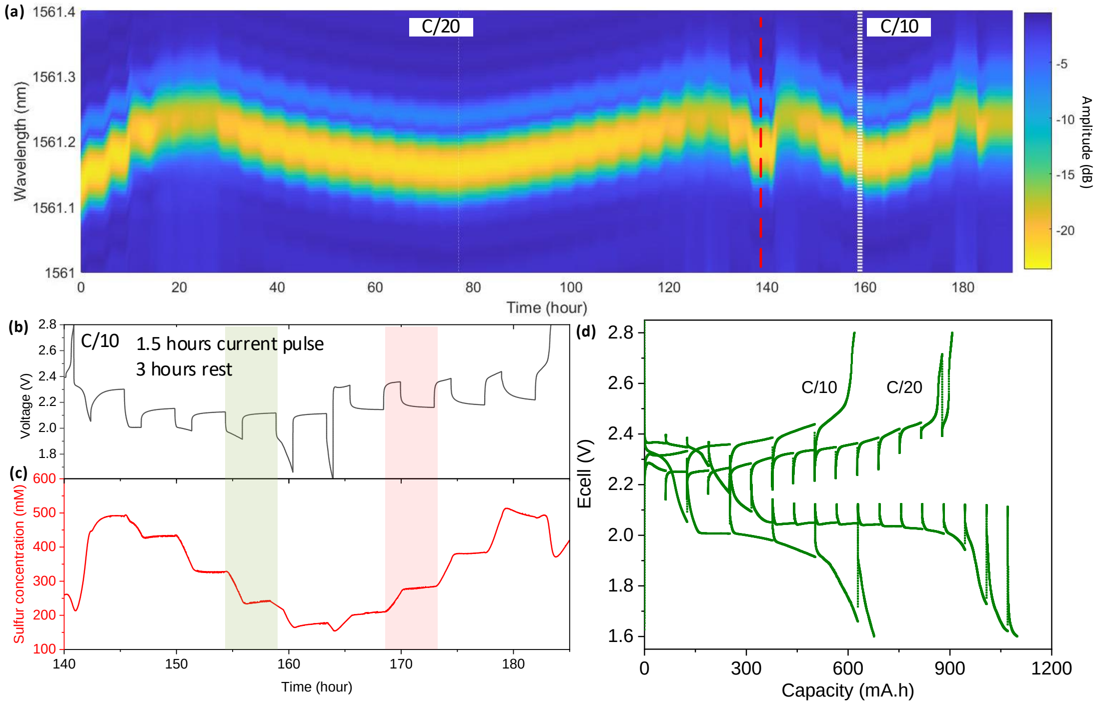

Fig. S8 | The optical spectra corresponding to GITT test (1.5 hours charge or discharge followed by 3 hours rest) at the cycling rate of C/20 and C/10 in a 25 C° oven. (b and c) The temporal voltage and decoded sulfur concentration of electrolyte during GITT at cycling rate of C/10. (d) The corresponding electrochemical performance of (a).

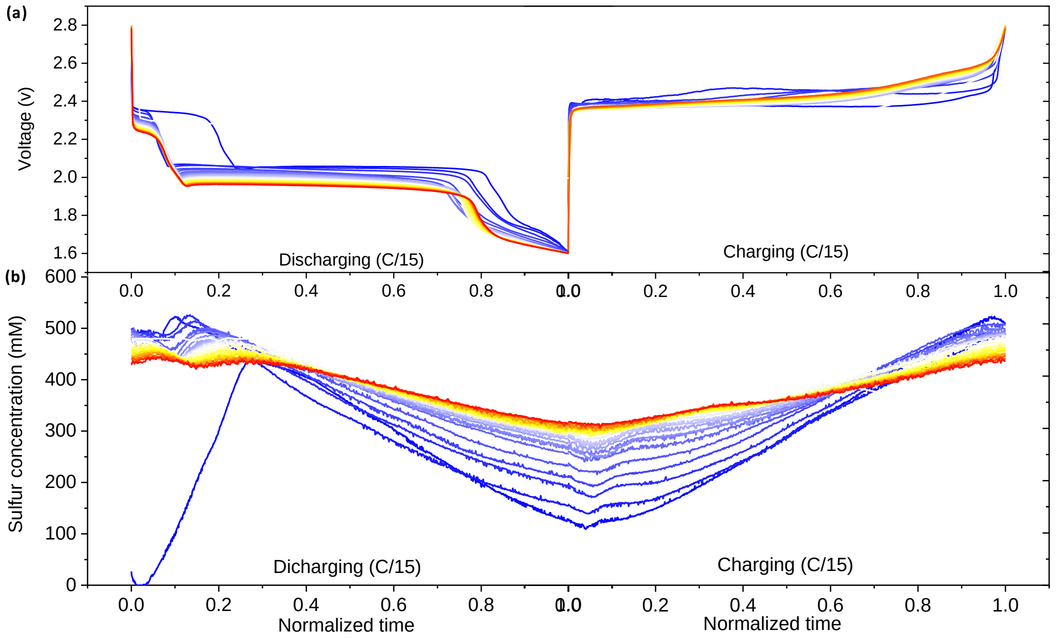

Fig. S9 | The time normalized temporal voltage (a) and decoded sulfur concentration (b) considering cathode made by super P and sulfur composite (27 cycles). As expected, the formation capability of Li_2S (the maximum sulfur concentration variation) and sulfur re-crystallization (the sulfur concentration decreasing at the end of charge) is fading when cycling.

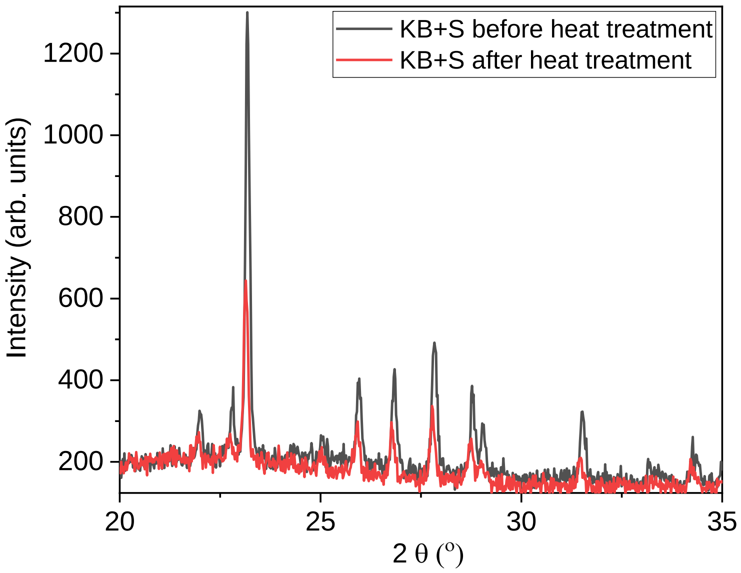

Fig. S10 | The XRD pattern before and after heat treatment of KB and sulfur. Partial sulfur is penetrating nanostructure of KB since the XRD peak of sulfur is not fully disappeared.

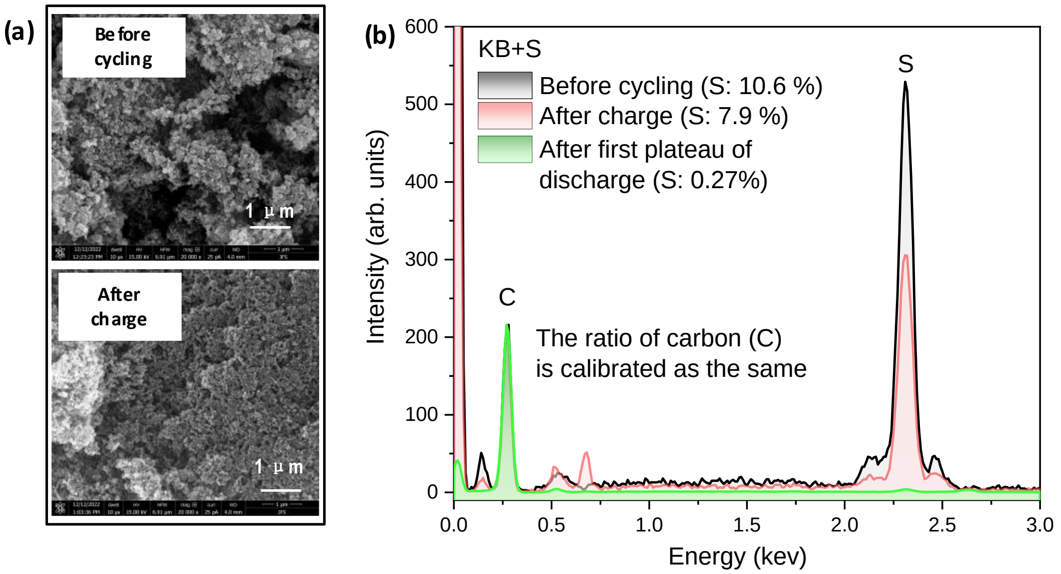

Fig. S11 | (a) Morphology (SEM) of the KB and sulfur cathode before cycling and after charge. (b) The quantitative analysis of sulfur before cycling, end of first plateau of discharge and end of charge.

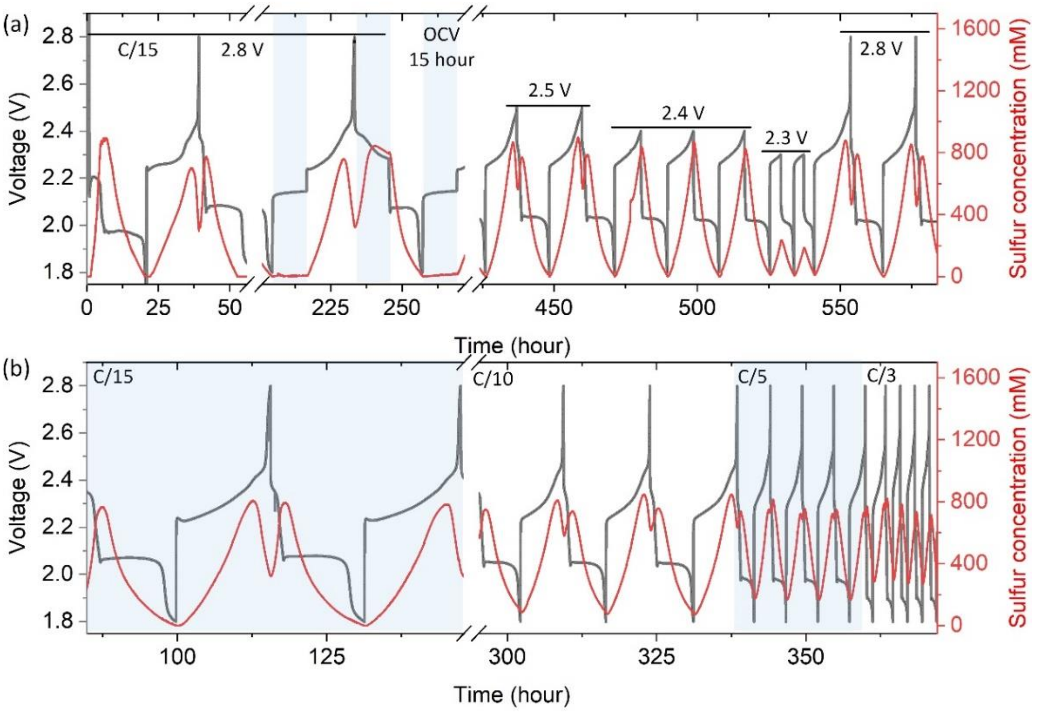

Fig. S12 | Operando measurement based on cathode with KB substrate. (a) The temporal voltage (black line) and decoded sulfur concentration of electrolyte (red line) for case 1: rest 15 hours after charging (no chemical reaction happens together with a constant sulfur concentration) and discharging (comproportionation reactions and solid sulfur transforms to polysulfide gradually, leading to sulfur concentration rise); case 2: two sulfur concentration peaks at the end (beginning) of charge (discharge) are related to sulfur recrystallization and dissolution. When decreasing the cutoff voltage, the depth of the two-peak valley is getting shallower (less sulfur crystallization) and finally disappear if the potential is equal or lower than 2.4 V. Apparently, it appears again if setting the cutoff voltage back to 2.8 V. (b) The cycling rate dependent of soluble sulfur concentration. The capability of Li_2S and sulfur crystallization becomes weak with higher cycling rate.

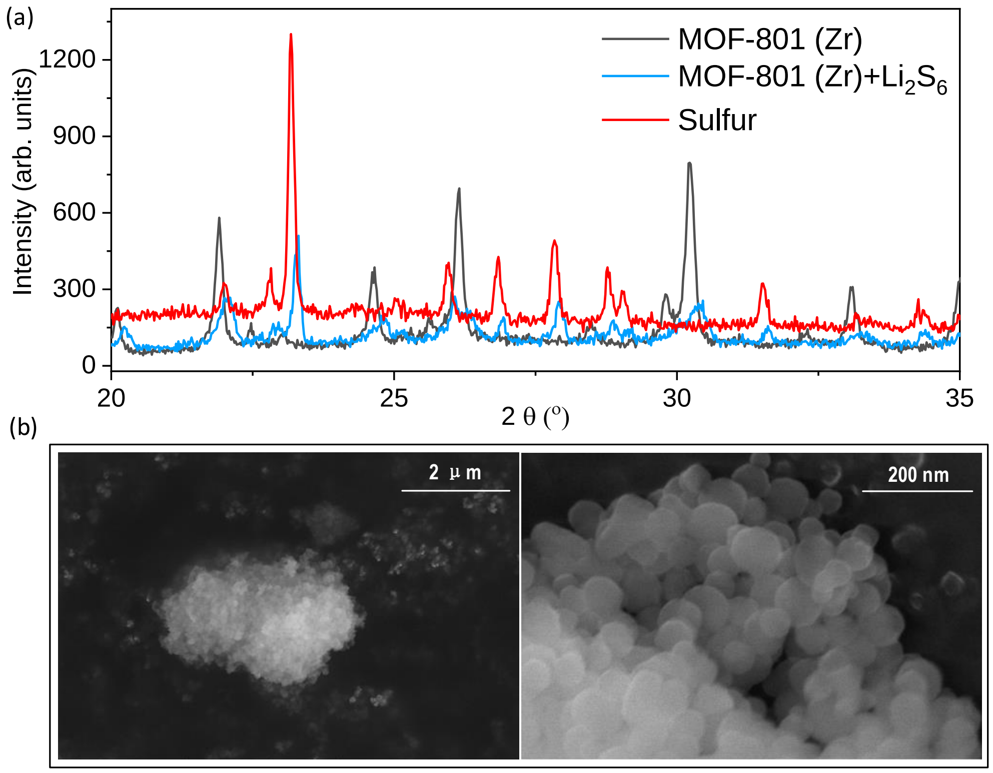

Fig. S13 | The characteristics of MOF-801(Zr) and polysulfide adsorption. (a) The XRD pattern of MOF-801(Zr) before (black line) and after (blue line) Li_2S_6 solution adsorption. The new peaks appear, matching to the peaks of crystal sulfur (red line). (b) The SEM of MOF-801(Zr).

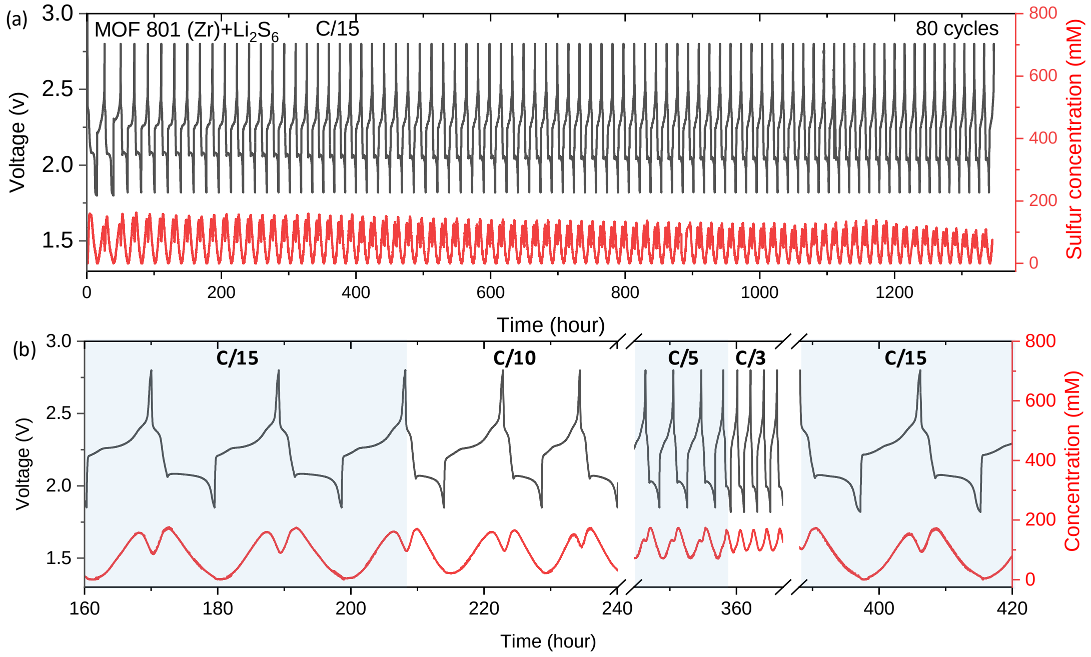

Fig. S14 | Operando measurement based on cathode with MOF-801(Zr) and super P substrate. (a) The temporal voltage (black line) and decoded sulfur concentration of electrolyte (red line) for 80 cycles. The crystallization of Li_2S and sulfur (sulfur concentration variation) is quite stable, which is the necessary conditions for long cycling. (b) The cycling

rate dependent of soluble sulfur concentration. The capability of Li_2S and sulfur crystallization becomes weak with higher cycling rate due to electrochemistry polarization.

Supplementary References

1. Albert, J., Shao, L. Y. & Caucheteur, C. Tilted fiber Bragg grating sensors. *Laser Photonics Rev.* **7**, 83–108 (2013).

2. Liu, F., Qi, Mei., Guo, T. & Albert, J. Saturable absorption and bistable switching of single mode fiber core-guided light by a 6 nm-thick, few layers graphene coating on the cladding surface. *Ann. Phys. (Berlin)* **532**, 2000157 (2020).

3. Chan, C.-F., Chen, C., Jafari, A., Laronche, A., Thomson, D. J. & Albert, J. Optical fiber refractometer using narrowband cladding-mode resonance shifts. *Appl. Opt.* **46**, 1142-1149 (2007).

4. Imas, J. J., Bai, X., Zamarreño, C. R., Matías, I. R. & Albert, J. Accurate compensation and prediction of the temperature cross-sensitivity of tilted FBG cladding mode resonance. *Appl. Opt.* **62**, E8-E15 (2023).

5. Cheon, S.-E., Ko, K.-S., Cho, J.-H., Kim, S.-W., Chin, E.-Y. & Kim, H.-T. Rechargeable lithium sulfur battery II. rate capability and cycle characteristics. *J. Electrochem. Soc.* **150**, A800-A805 (2003).

6. Jozwiuk, A., Berkes, B. B., Weiβ, T., Sommer, H., Janek, J. & Brezesinski, T. The critical role of lithium nitrate in the gas evolution of lithium-sulfur batteries. *Energy Environ. Sci.* **9**, 2603-2608 (2016).
