# Supplementary material for: Detangling electrolyte chemical dynamics in lithium sulfur batteries by operando monitoring with optical resonance combs
Source: Nat Commun. 2023 Nov 14;14:7350. doi: 10.1038/s41467-023-43110-8 (PMC10645864; doi:10.1038/s41467-023-43110-8)
Supplement: Supplementary file 3 — Description of Additional Supplementary Files [file 41467_2023_43110_MOESM3_ESM.docx]

File Name: Supplementary Movie 1
Description: Li-S battery TFBG sensing over cycling
